# Supplementary material for: A fault-intrusion-tolerant system and deadline-aware algorithm for scheduling scientific workflow in the cloud
Source: PeerJ Comput Sci. 2021 Nov 2;7:e747. doi: 10.7717/peerj-cs.747 (PMC8576554; doi:10.7717/peerj-cs.747)
Supplement: Supplemental Information 6 [file peerj-cs-07-747-s006.zip › Myproject1.1/docs/org/cloudbus/cloudsim/examples/CloudSimExample4.html]

CloudSimExample4


JavaScript is disabled on your browser.


- Overview
- Package
- Class
- Use
- Tree
- Deprecated
- Index
- Help

- Prev Class
- Next Class

- Frames
- No Frames

- All Classes

- Summary:
- Nested |
- Field |
- Constr |
- Method

- Detail:
- Field |
- Constr |
- Method


org.cloudbus.cloudsim.examples

## Class CloudSimExample4

- java.lang.Object
- - org.cloudbus.cloudsim.examples.CloudSimExample4

- ---

    

  ```
  public class CloudSimExample4
  extends java.lang.Object
  ```

  A simple example showing how to create
  two datacenters with one host each and
  run two cloudlets on them.

- - ### Constructor Summary

    Constructors

    Constructor and Description

    `CloudSimExample4()`
  - ### Method Summary

    Methods

    | Modifier and Type | Method and Description |
    | --- | --- |
    | `static void` | `main(java.lang.String[] args)` Creates main() to run this example |

    - ### Methods inherited from class java.lang.Object

      `clone, equals, finalize, getClass, hashCode, notify, notifyAll, toString, wait, wait, wait`

- - ### Constructor Detail


    - #### CloudSimExample4

      ```
      public CloudSimExample4()
      ```
  - ### Method Detail


    - #### main

      ```
      public static void main(java.lang.String[] args)
      ```

      Creates main() to run this example


- Overview
- Package
- Class
- Use
- Tree
- Deprecated
- Index
- Help

- Prev Class
- Next Class

- Frames
- No Frames

- All Classes

- Summary:
- Nested |
- Field |
- Constr |
- Method

- Detail:
- Field |
- Constr |
- Method
